# The Synergistic Interaction Between BAP and GA3 Eradicates Hyperhydricity and Enhances Biomass in *Solanum tuberosum* L. cv. ‘Superchola’ In Vitro Plantlets

**DOI:** 10.3390/plants15193071

**Published:** 2026-10-08

**Authors:** Guillermo Alexander Jácome Sarchi, Stalin Aldair De la Cruz Sarchi, Jorge Ivan Mina Ortega, Francisca Hernández, Rafael Todos Santos Martínez

**Affiliations:** 1 Grupo de Investigación Agricultura Sostenible (GIAS), Carrera de Agropecuaria, Facultad de Industrias Agropecuarias y Ciencias Ambientales, Universidad Politécnica Estatal del Carchi, Tulcán 040102, Ecuador; 2 Carrera de Alimentos, Facultad de Industrias Agropecuarias y Ciencias Ambientales, Universidad Politécnica Estatal del Carchi, Tulcán 040102, Ecuador; stalin.delacruz@upec.edu.ec (S.A.D.l.C.S.); jorge.mina@upec.edu.ec (J.I.M.O.); 3 Grupo de Investigación en Fruticultura y Técnicas de Producción, Instituto de Investigación e Innovación Agroalimentaria y Agroambiental (CIAGRO-UMH), Universidad Miguel Hernández, 03312 Orihuela, Spain; francisca.hernandez@umh.es

**Keywords:** pre-basic seed potato, micropropagation, plant growth regulator, vitrification, morphological vigor, Andean cultivars

## Abstract

The mass propagation of pre-basic seed potatoes (*Solanum tuberosum* L.) is frequently bottlenecked by the physiological degradation of in vitro plantlets, largely driven by cytokinin oversaturation. This study evaluated the morphogenic and physiological effects of the synergistic interaction between 6-Benzylaminopurine (BAP) and Gibberellic Acid (GA3) on the Andean cultivar ‘Superchola’. Using a completely randomized 3 × 3 factorial design, nodal segments were cultured on Murashige and Skoog (MS) medium supplemented with BAP (0.5, 1.0, 2.0 mg/L) and GA3 (0.0, 0.1, 0.25 mg/L) for 28 days. The results demonstrated that while moderate BAP concentrations (1.0 mg/L) maximized the absolute shoot multiplication rate, they simultaneously induced severe hyperhydricity (71.2%) and basal callogenesis (80.0%) when GA3 was deficient. Conversely, a strict synergistic interaction comprising 2.0 mg/L BAP and 0.1 mg/L GA3 completely eradicated hyperhydricity (0.0%), restricted callogenesis (5.0%), and yielded plantlets with the highest Morphological Vigor Index (81.2% elite classification). Furthermore, this specific hormonal balance maximized structural dry biomass accumulation (95 mg/explant) and normalized the fresh-to-dry weight ratio, proving the elimination of deceptive water retention. These findings confirm that an optimum BAP/GA3 combination is critical to suppressing physiological disorders during the in vitro multiplication phase.

## 1. Introduction

The mass production of high-quality pre-basic seed potatoes (*Solanum tuberosum* L.) relies heavily on in vitro micropropagation technologies and advanced tuberization systems [1]. For Andean cultivars of high agronomic value, such as ‘Superchola’, establishing efficient organogenic scaling protocols is critical to meet the demands of global food security and local seed production [2,3]. However, the transition from in vitro biofactories to ex vitro greenhouse conditions remains a major bottleneck [4]. The survival and agronomic performance of these clones during acclimatization strictly depend on the morphological vigor and physiological competence acquired during the in vitro multiplication phase [4]. Consequently, forcing rapid shoot proliferation often compromises the structural integrity of the plantlets, leading to massive losses due to transplant shock during ex vitro transfer [4,5].

To overcome genotype-specific recalcitrance and maximize multiplication rates, commercial tissue culture protocols for potato frequently employ high concentrations of cytokinins, particularly 6-benzylaminopurine (BAP) [6,7]. While effective in breaking apical dominance and stimulating axillary meristem activation, cytokinin oversaturation frequently induces severe physiological disorders in *Solanum tuberosum* L. explants. Prolonged exposure to unbalanced BAP levels disrupts normal lignification and cell differentiation, triggering tissue vitrification (hyperhydricity) and cellular dedifferentiation (basal callogenesis) [8,9]. These anomalies not only drastically reduce the morphological vigor of the clones but also render them highly susceptible to oxidative stress and necrosis. Thus, achieving a high multiplication rate is biologically futile if the resulting plantlets lack the structural fidelity required for successful greenhouse acclimatization. Recently, automated platforms such as Temporary Immersion Bioreactors (TIBs) have emerged as highly efficient, hormone-free alternatives to mitigate hyperhydricity in potato micropropagation by controlling physical contact with liquid media [1]. However, the high initial capital investment, technical maintenance, and operational complexity of these pneumatic systems restrict their massive adoption in local Andean biofactories. Consequently, optimizing conventional semi-solid systems through a highly calibrated chemical protocol remains an economically viable and strictly necessary strategy to overcome the recalcitrance of Andean ecotypes like ‘Superchola’.

Supporting this chemical approach, recent models in potato micropropagation suggest that morphological stability in highly morphogenic environments can be restored through precise synergistic interaction [6]. Gibberellic acid (GA3), known for driving internodal elongation and cell distension, could act antagonistically to mitigate BAP-induced physiological stress, provided the concentration ratios are strictly calibrated [10]. Therefore, this study hypothesizes that a synergistic balance between BAP and low-dose GA3 can suppress hyperhydricity and callogenesis while maximizing shoot multiplication and dry biomass accumulation. To test this, we evaluated the effects of various cytokinin–gibberellin combinations on shoot multiplication and their morphology on *Solanum tuberosum* L. ‘Superchola’, aiming to establish a mechanistic protocol that guarantees both massive proliferation and optimal morphological vigor in pre-basic seed potato production platforms.

## 2. Results

### 2.1. Shoot Multiplication

**Figure 1:**
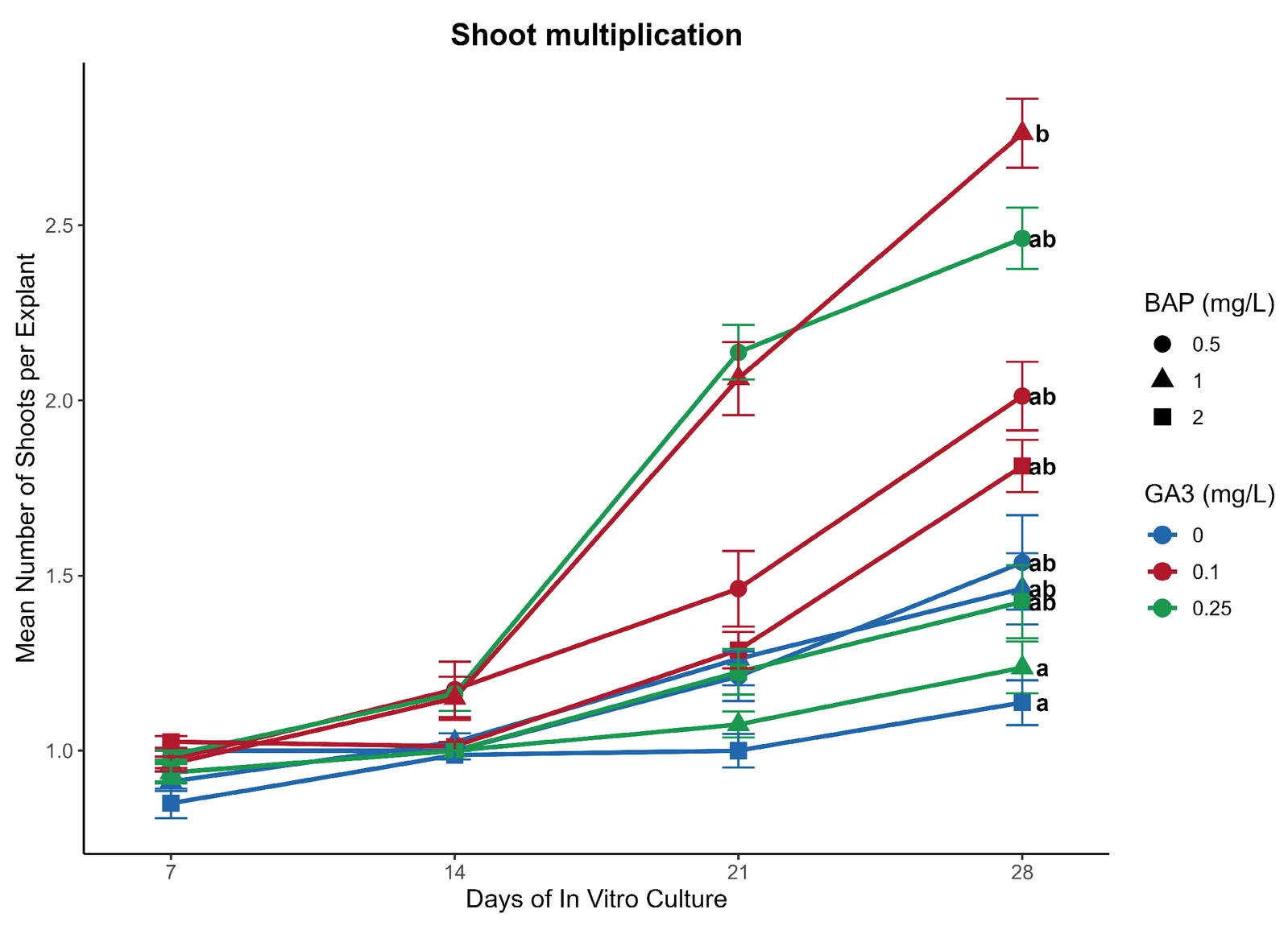
Shoot multiplication of *Solanum tuberosum* L. over 28 days of in vitro culture. Data points represent the mean number of shoots per explant under different BAP and GA3 interactions. Error bars represent the standard error of the mean (n = 20). Different letters above the bars indicate significant differences according to Tukey’s multiple comparison test derived from a Binomial Generalized Linear Model (*p* < 0.05).

The temporal progression of shoot multiplication over the 28-day in vitro culture period revealed highly significant interaction effects between BAP and GA3 (Figure 1; Table S2). Initial morphogenic responses (Day 7) were relatively homogeneous across all hormonal combinations. However, exponential shoot proliferation was triggered after Day 14, specifically in explants subjected to low-to-intermediate BAP concentrations supplemented with GA3. By Day 28, the treatments comprising 1.0 mg/L BAP + 0.1 mg/L GA3 and 0.5 mg/L BAP + 0.25 mg/L GA3 exhibited the steepest kinetic slopes, peaking at maximum multiplication rates (Figure 1).

Conversely, treatments lacking GA3 (0.0 mg/L), particularly when combined with high cytokinin levels (2.0 mg/L BAP), severely restricted shoot proliferation, maintaining a nearly flat kinetic curve throughout the evaluation period.

### 2.2. Suppression of Hyperhydricity

**Figure 2:**
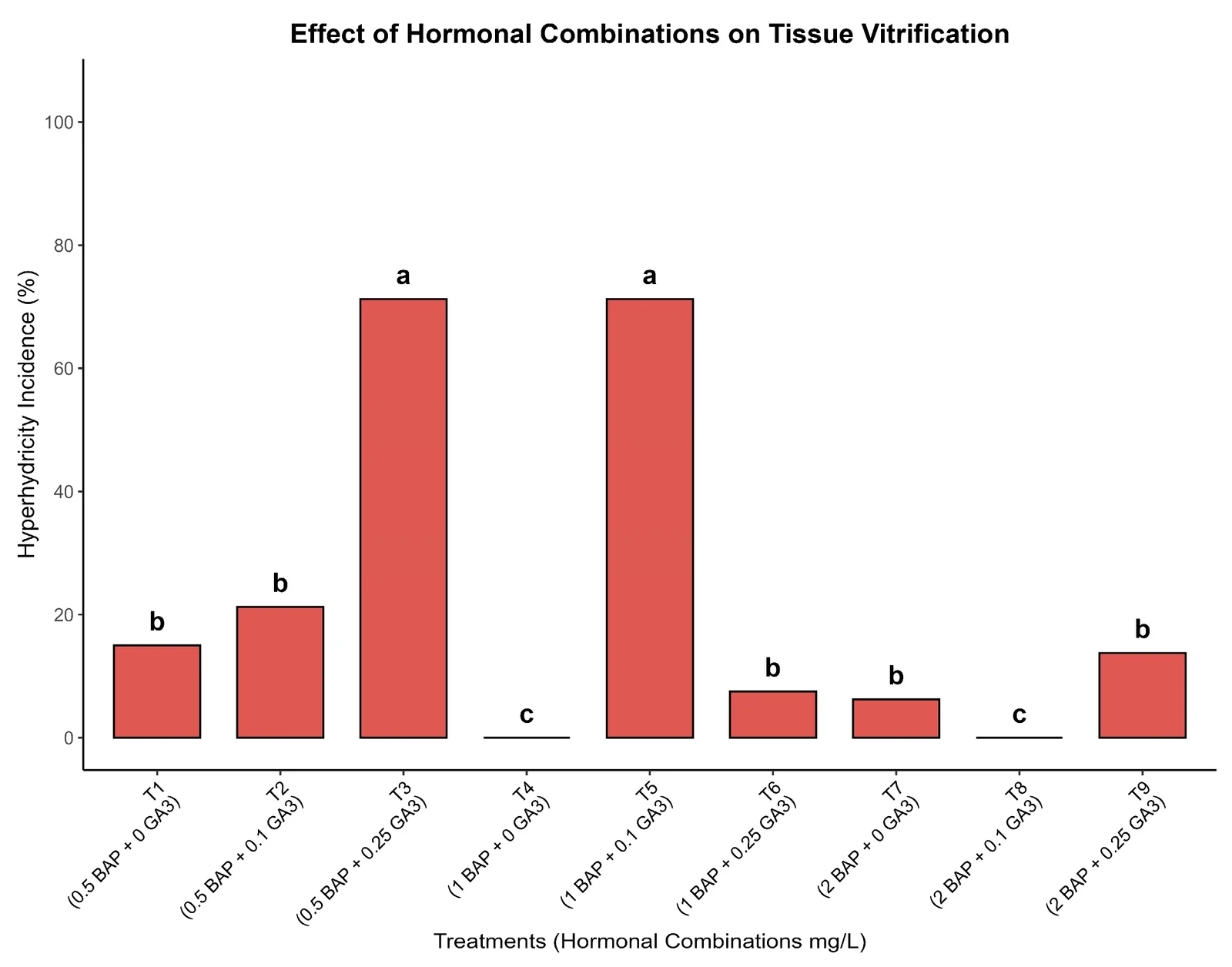
Effect of hormonal combinations on hyperhydricity. Bars represent the incidence percentage of hyperhydric explants of *Solanum tuberosum* L. after 28 days of culture. Different letters above the bars indicate significant differences according to Tukey’s multiple comparison test derived from a Binomial Generalized Linear Model (*p* < 0.05).

**Figure 3:**
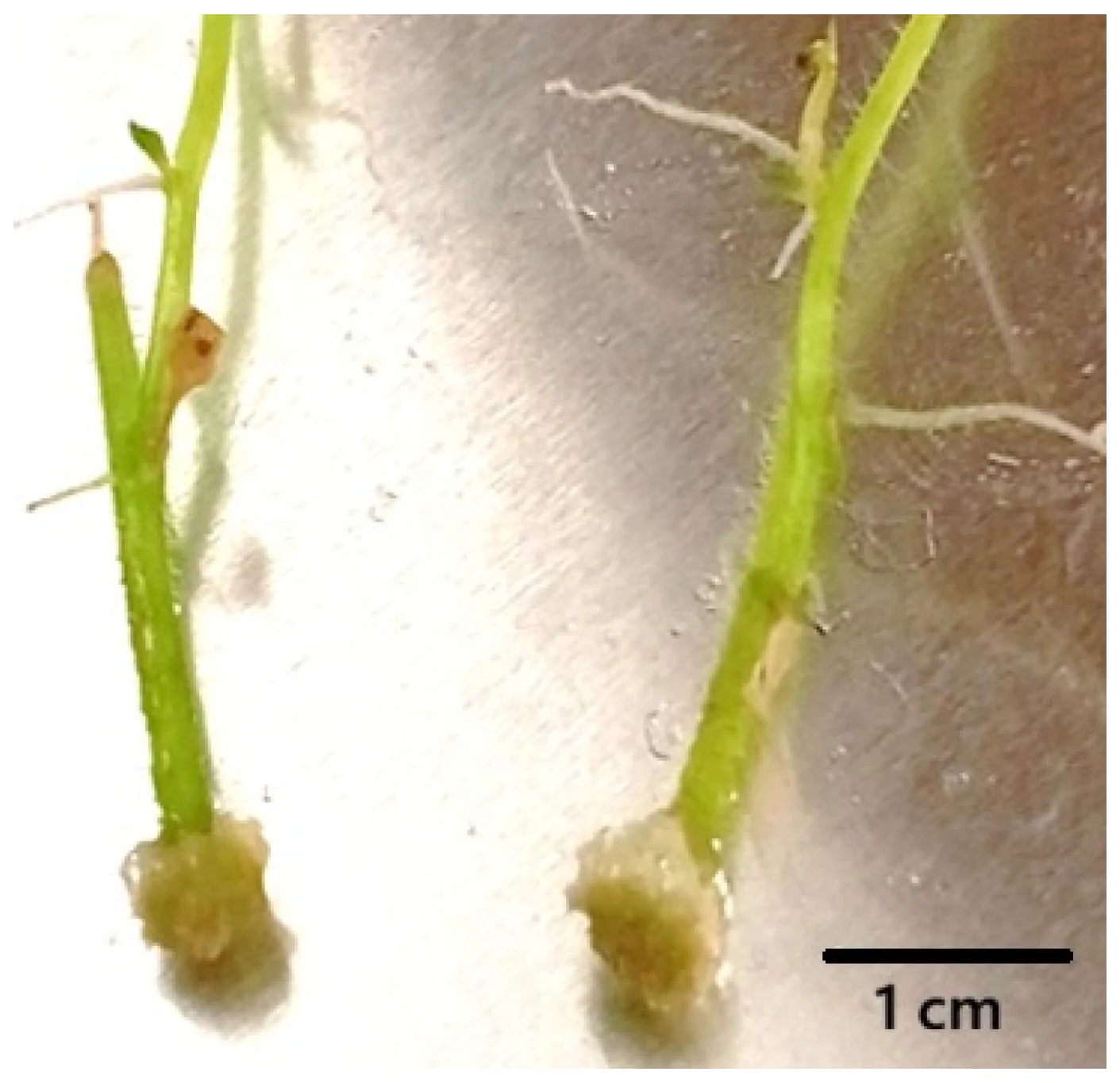
Representative phenotype of physiological disorders observed in *Solanum tuberosum* L. cv. ‘Superchola’ explants after 28 days of in vitro culture under unbalanced BAP/GA3 hormonal ratios (specifically 0.5 mg/L BAP + 0.25 mg/L GA3). The image highlights amorphous basal callogenesis and structural tissue degradation, which act as primary physical indicators of morphogenic instability prior to severe hyperhydricity.

The hormonal combinations significantly influenced the morphological stability of the in vitro explants (Figure 2, corresponding to the physical phenotypes illustrated in Figure 3). Explants subjected to unbalanced hormonal ratios, specifically 0.5 mg/L BAP + 0.25 mg/L GA3 (T3) and 1.0 mg/L BAP + 0.1 mg/L GA3 (T5), exhibited a drastic increase in tissue hyperhydricity, reaching incidences of over 70% (*p* < 0.001). Conversely, treatments with optimized cytokinin–gibberellin ratios, notably 2.0 mg/L BAP + 0.1 mg/L GA3 (T8) and 1.0 mg/L BAP + 0.0 mg/L GA3 (T4), completely suppressed this physiological anomaly, demonstrating a 0.0% incidence of hyperhydricity.

### 2.3. Mitigation of Basal Callogenesis

**Figure 4:**
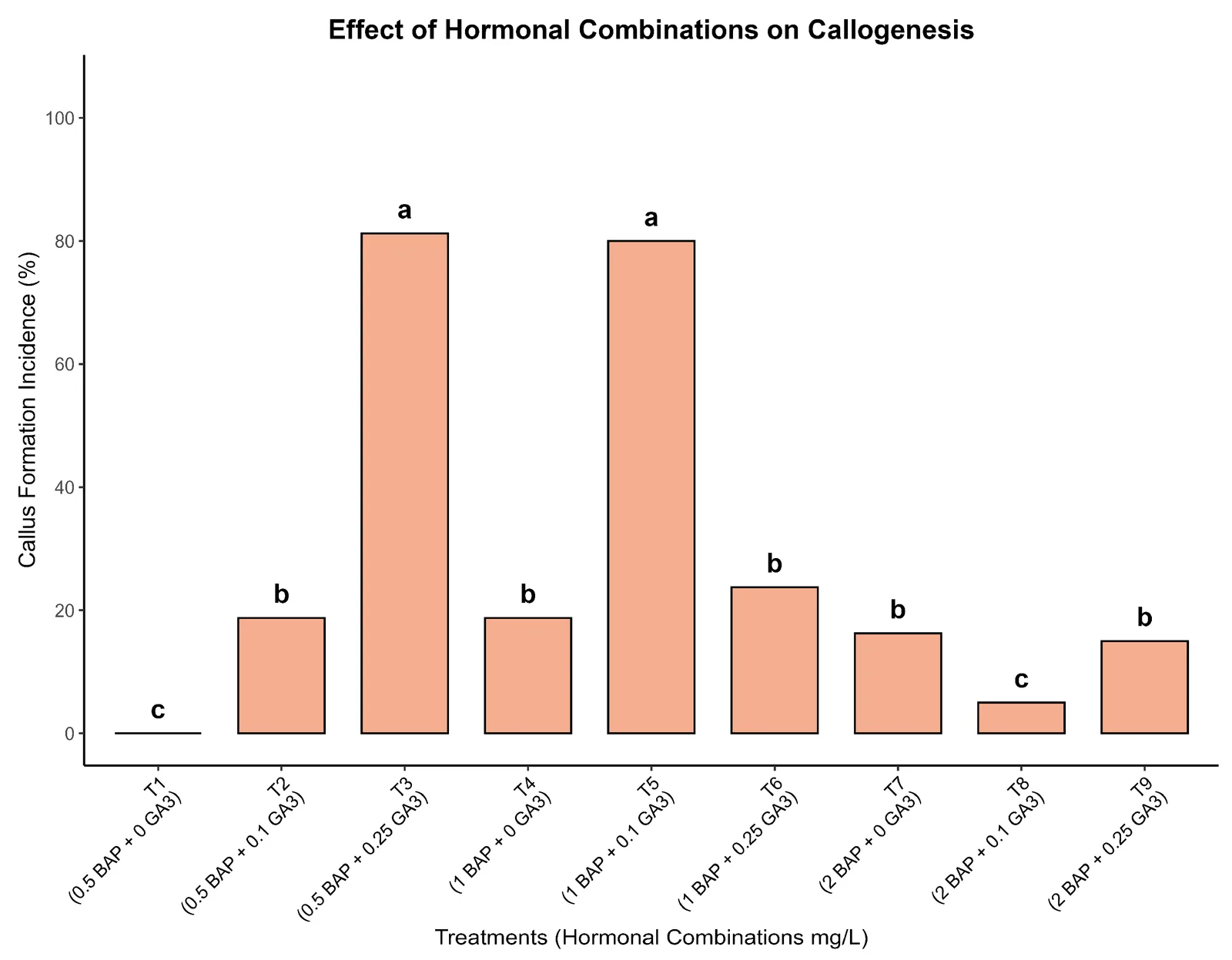
Effect of hormonal combinations on basal callogenesis. Bars represent the incidence percentage of callus formation. Different letters indicate significant differences according to Tukey multiple comparison test (*p* < 0.05).

Parallel trends regarding cellular dedifferentiation were observed (Figure 4). Unbalanced treatments that induced high vitrification (T3 and T5) also triggered excessive basal callogenesis, with incidences surpassing 80%. In contrast, the hormonal combination in treatment T8 (2.0 mg/L BAP + 0.1 mg/L GA3) restricted callus formation to merely 5.0%.

### 2.4. Enhancement of Morphological Vigor

**Figure 5:**
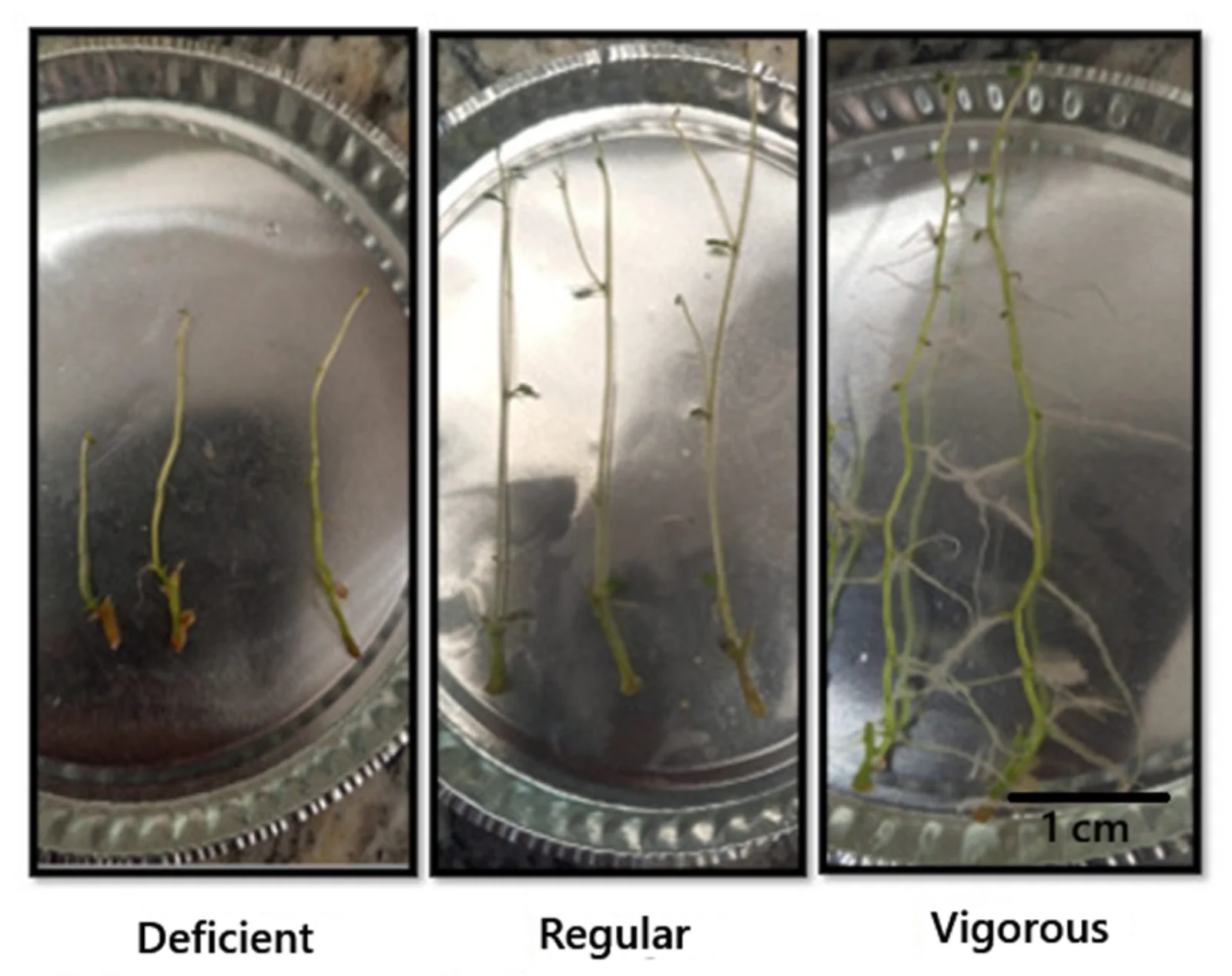
Visual classification scale for the Morphological Vigor Index (IVM) applied to micropropagated *Solanum tuberosum* L. cv. ‘Superchola’ plantlets after 28 days of culture: (1) Deficient, marked by stunted growth, micro-shoots, and basal necrosis; (2) Regular, showing standard stem elongation with moderate robustness; and (3) Vigorous, denoting elite structural integrity, well-developed root systems, and balanced stem thickness achieved under the optimized hormone combination (Treatment T8: 2.0 mg/L BAP + 0.1 mg/L GA3). Scale bars = 1 cm.

**Figure 6:**
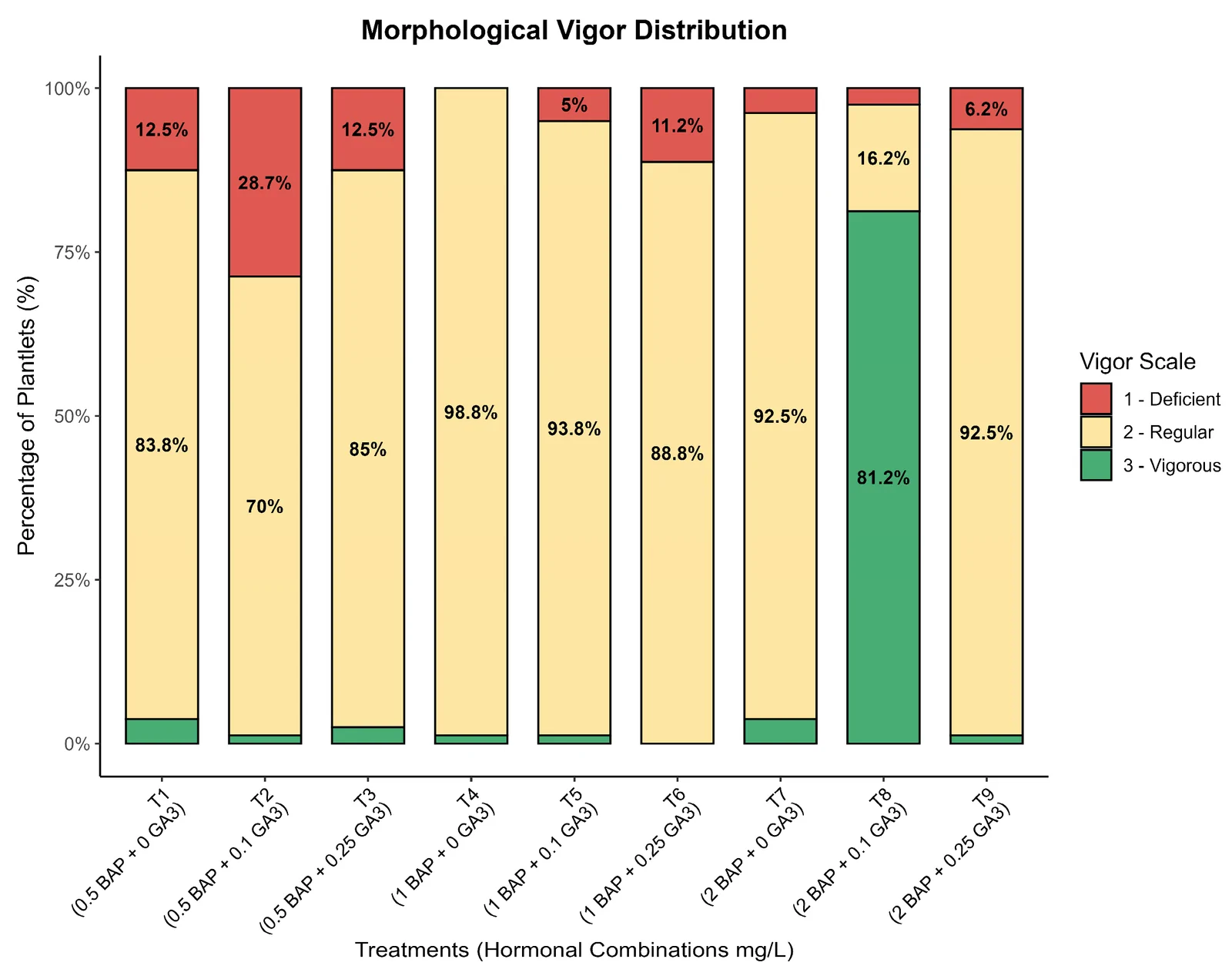
Distribution of the Morphological Vigor Index across different hormonal treatments. Stacked bars represent the percentage of *Solanum tuberosum* L. plantlets categorized as Deficient, Regular, or Vigorous after 28 days of in vitro culture (n = 80 explants per treatment).

The qualitative assessment of the Morphological Vigor Index (1 = Deficient, 2 = Regular, 3 = Vigorous; Figure 5) revealed that hormonal interaction critically dictates the structural quality of the in vitro plantlets, an essential parameter for subsequent ex vitro acclimatization (Figure 6). The vast majority of treatments induced a predominantly ‘Regular’ structural phenotype. However, treatment T8 (2.0 mg/L BAP + 0.1 mg/L GA3) significantly outperformed all other hormonal combinations, yielding 81.2% of plantlets with the highest ‘Vigorous’ classification. Conversely, treatments with imbalanced ratios, such as T1 and T2, exhibited noticeable percentages of ‘Deficient’ plantlets (12.5% and 28.7%, respectively).

### 2.5. Results—Dry Biomass and Water Dynamics

**Figure 7:**
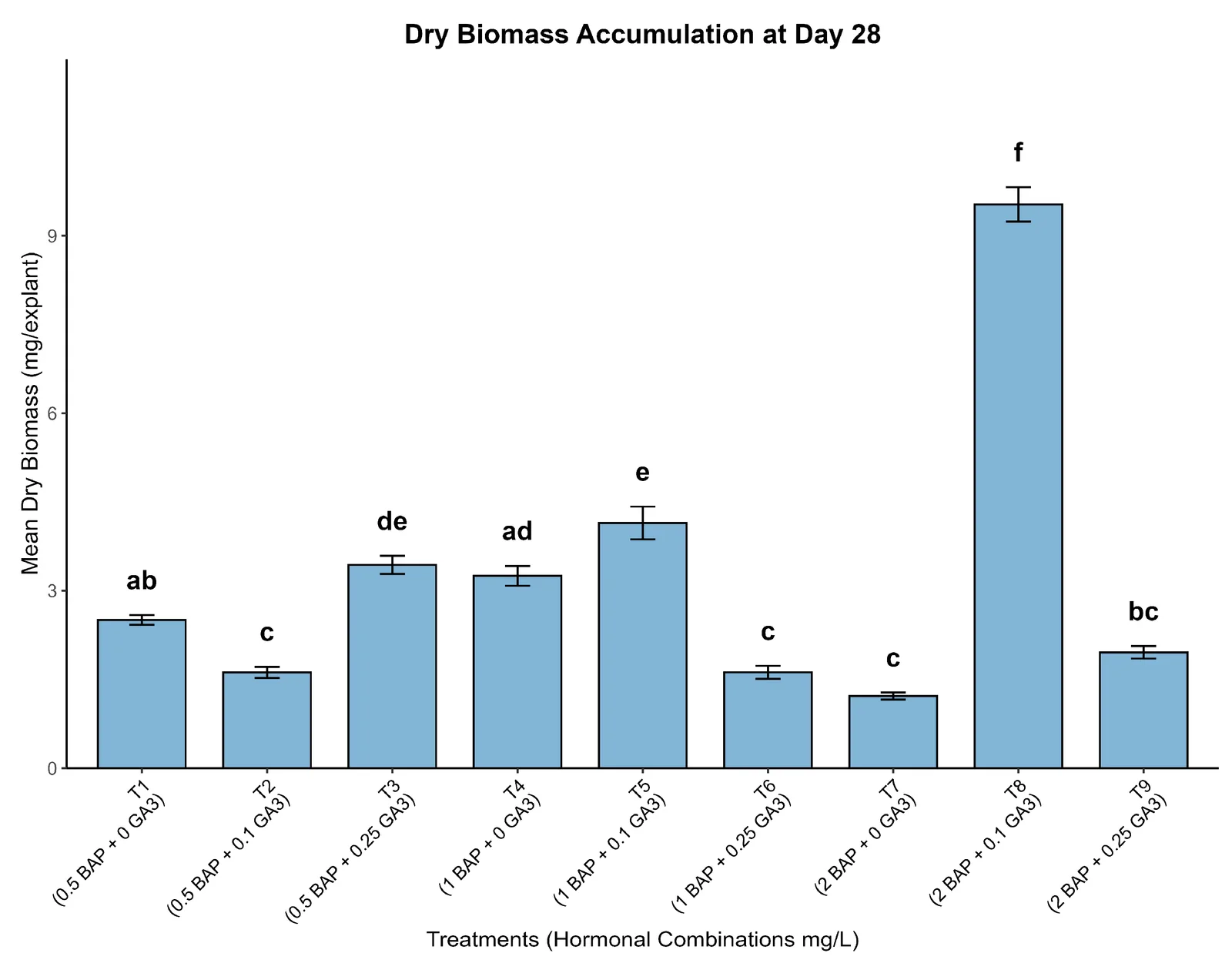
Dry biomass accumulation of *Solanum tuberosum* L. explants after 28 days of in vitro culture under different hormonal interactions. Bars represent the mean dry weight (mg) per explant. Error bars indicate the standard error of the mean (n = 20). Different letters above the error bars denote significant differences according to Tukey’s honest significant difference test (*p* < 0.05).

**Figure 8:**
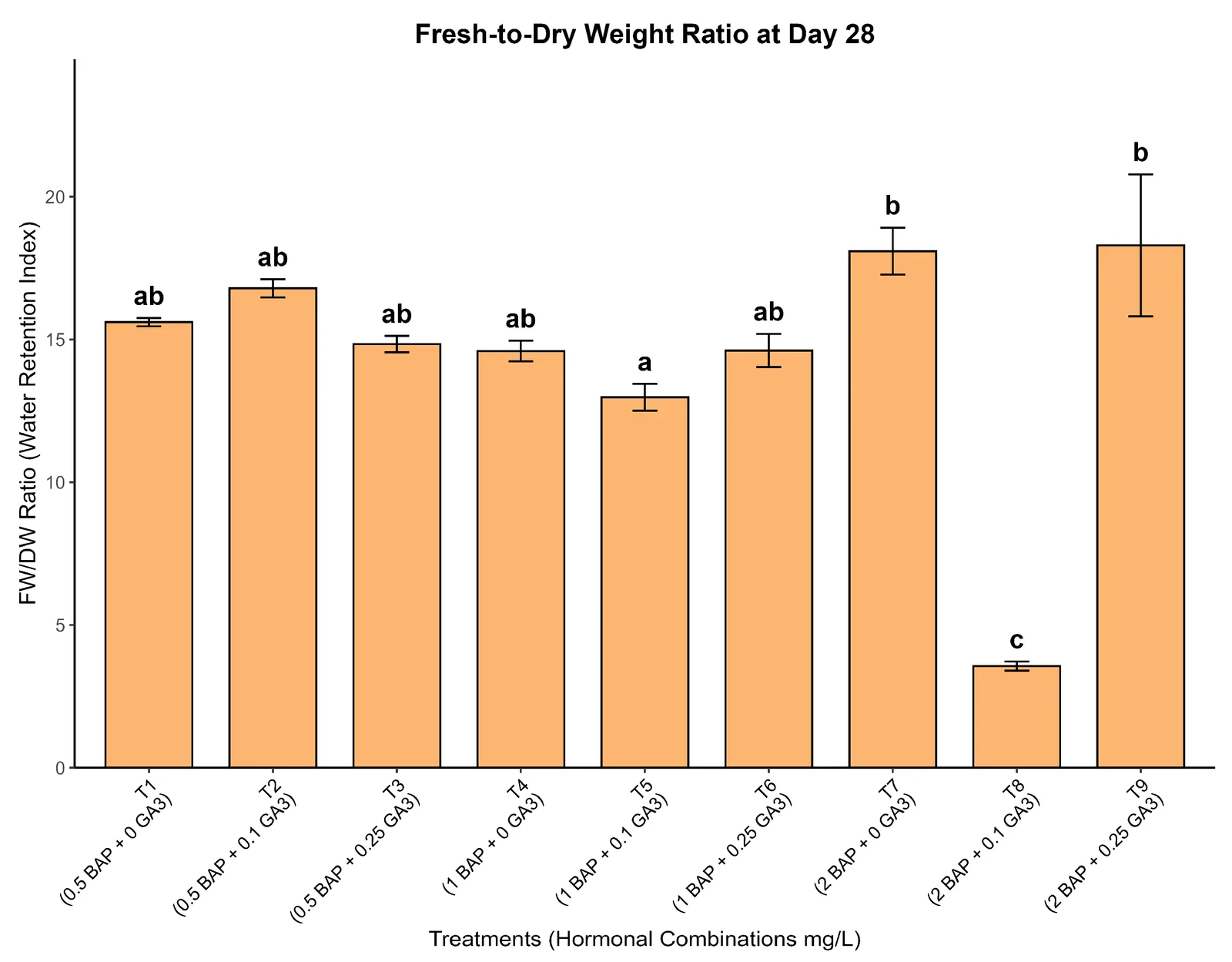
Fresh-to-dry weight (FW/DW) ratio of *Solanum tuberosum* L. cv. ‘Superchola’ explants after 28 days of in vitro culture under different BAP and GA3 hormonal interactions. Bars represent the mean FW/DW ratio (water retention index) per explant. Error bars indicate the standard error of the mean (n = 20). Different letters above the error bars denote significant differences according to Tukey’s honest significant difference test (*p* < 0.05).

To confirm that the observed morphogenic responses translated into actual structural tissue rather than deceptive water retention, dry biomass accumulation, fresh weight (FW), and fresh-to-dry weight (FW/DW) ratios were quantified at the end of the 28-day culture cycle (Figure 7 and Figure 8). Analysis of variance revealed highly significant differences among the hormonal interactions (*p* < 0.001; Table S1). Consistent with the qualitative assessments of structural vigor, treatment T8 (2.0 mg/L BAP + 0.1 mg/L GA3) maximized dry matter accumulation, achieving a peak mean dry biomass of 95 mg per explant, which significantly outperformed all other hormonal combinations (*p* < 0.05). Conversely, treatments exhibiting severe hyperhydricity (e.g., T3 and T5) displayed inflated fresh weights exceeding 50 mg and high FW/DW ratios (averaging 14.84 and 12.97, respectively, Figure 8), confirming excessive intercellular water accumulation despite high initial cell proliferation. Furthermore, the optimized treatment T8 successfully normalized the FW/DW ratio down to 3.56 (Figure 8), quantitatively validating the complete elimination of vitrification and true lignocellulosic tissue consolidation. Finally, while treatment T9 (2.0 mg/L BAP + 0.25 mg/L GA3) exhibited low hyperhydricity, its slight excess in gibberellins shifted metabolic energy toward excessive internodal elongation (etiolation) rather than structural reinforcement, resulting in structurally fragile stems and a severe penalty on total dry biomass.

## 3. Discussion

The optimization of in vitro multiplication protocols for recalcitrant Andean potato cultivars, such as ‘Superchola’, requires a delicate balance between maximizing shoot proliferation and maintaining absolute structural fidelity [11,12,13,14]. The results of this study clearly demonstrate that while cytokinin supplementation is obligatory for breaking apical dominance, its isolated or unbalanced application severely compromises plantlet viability. Our findings support the hypothesis that a precise synergistic interaction between BAP and GA3 is the primary driver of both high multiplication rates and optimal morphological vigor, effectively suppressing physiological disorders that typically hinder ex vitro acclimatization [15,16,17].

### 3.1. Synergistic Interaction in Shoot Proliferation

The shoot multiplication (Figure 1) revealed that BAP acts as the primary catalyst for meristematic activation. However, treatments relying heavily on BAP without adequate GA3 compensation exhibited delayed or suppressed kinetic slopes. Previous studies have documented that high concentrations of cytokinins can induce an auto-inhibitory feedback loop, downregulating the expression of endogenous gibberellin biosynthesis genes and altering shoot apical meristem formation [18,19,20]. In our study, the interaction of 2.0 mg/L BAP with a low dose of 0.1 mg/L GA3 (Treatment T8) generated a steady, vigorous proliferation rate. Interestingly, while T5 (1.0 mg/L BAP + 0.1 mg/L GA3) produced the highest absolute number of shoots, this metric alone was deceptive, as it was accompanied by severe physiological degradation, corroborating recent reports that hyper-proliferation induced by excess cytokinins often comes at the cost of structural tissue quality [21,22,23]. This morphogenic paradox demonstrates that hyperproliferation and tissue toxicity are driven by molar stoichiometric imbalances between cytokinins and gibberellins, rather than the absolute concentration of BAP alone.

### 3.2. Suppression of Physiological Disorders

A key finding in this study is the quantified suppression of hyperhydricity and basal callogenesis through hormonal antagonism. Hyperhydricity is a severe physiological disorder driven by oxidative stress, impaired stomatal function, and excessive water retention, frequently triggered by cytokinin oversaturation in closed environments [24,25,26,27]. Our data (Figure 2, complemented by the physical phenotypes in Figure 3) showed that unbalanced ratios (e.g., T3 and T5) resulted in over 70% vitrification. Notably, the elevation of BAP to 2.0 mg/L, when strictly paired with 0.1 mg/L GA3 (T8), completely eradicated hyperhydricity (0.0%). The absence of vitrification in T4 (1.0 mg/L BAP alone) indicates that this moderate cytokinin level does not cross the toxicity threshold; however, unbalanced GA3 addition (T5) collapses this equilibrium. This suggests that maintaining a strict stoichiometric ratio, especially at higher proliferation thresholds (2.0 mg/L BAP), allows GA3 to act as a physiological buffer, potentially promoting proper cell wall lignification and mitigating cytokinin-induced oxidative stress [23,28]. Parallel trends were observed in callogenesis (Figure 4), where the T8 formulation restricted cellular dedifferentiation to a mere 5.0%, maintaining the biomass allocation directed toward apical growth rather than amorphous callus formation [22,29].

### 3.3. Morphological Vigor and Biomass Accumulation

The ultimate determinant of micropropagation success is the physiological competence of the clones prior to greenhouse transfer [4]. The Morphological Vigor Index and dry biomass data definitively isolate T8 as the elite treatment. While GA3 is typically associated with internodal elongation, excessive doses frequently lead to undesirable etiolation and fragile stems. Here, the strict calibration of 0.1 mg/L GA3 prevented etiolation while allowing optimal cell distension, resulting in plantlets with excellent structural robustness (81.2% categorized as ‘Vigorous’, supported by the visual scale in Figure 5 and the distribution in Figure 6). Crucially, this vigor was mathematically validated by the dry biomass accumulation (Figure 7) and the normalized water dynamics (Figure 8). Treatment T8 yielded the highest accumulation of dry matter (0.0095 g per explant) and successfully collapsed the FW/DW ratio to a physiological baseline of 3.56. This confirms that the observed volume in T8 plantlets was constituted by actual lignocellulosic matrix and functional photosynthetic tissues [5], rather than the deceptive intracellular water accumulation characteristic of hyperhydric treatments (FW/DW > 12) [25,30].

## 4. Materials and Methods

### 4.1. Plant Material and Explant Preparation

Certified pathogen-free in vitro plantlets of *Solanum tuberosum* L. cultivar ‘Superchola’ were supplied by the National Institute of Agricultural Research (INIAP, Ecuador). Plantlets underwent a strictly controlled 28-day multiplication cycle to ensure optimal physiological vigor and cellular youth prior to the experiment [31,32]. Under strict aseptic conditions within a laminar flow hood, vigorous stems were excised to obtain homogeneous nodal segments of 1.0–1.5 cm in length [33]. The basal and apical portions of the source plantlets were systematically discarded to minimize physiological variability, and each selected explant contained at least one fully developed axillary bud.

### 4.2. Culture Media Composition and Sterilization

The basal culture medium was composed of 100% Murashige and Skoog (MS) salts and vitamins (PhytoTech Labs, Lenexa, KS, USA), supplemented with 30 g/L of sucrose as the primary carbon source [34]. To prevent endogenous microbial contamination without altering morphogenic responses, 1.0 mL/L of Plant Preservative Mixture (PPM™, Plant Cell Technology, Smithfield, UT, USA) was incorporated into the medium prior to sterilization [35]. The pH of the solution was strictly adjusted to 5.8 using 1N NaOH and 1N HCl with a digital benchtop pH meter (model HI2211, Hanna Instruments, Woonsocket, RI, USA). Finally, the medium was solidified with 7.0 g/L of Supreme TC Grade Agar (Plant Cell Technology, Smithfield, UT, USA). The prepared media were dispensed into glass culture vessels (approx. 25 mL per vessel) and autoclaved at 121 °C and 15 psi for 20 min using a vertical laboratory autoclave (model MLS-3781L, Panasonic Healthcare Co., Tokyo, Japan). The biological neutrality of this specific PPM concentration was previously established based on standard protocols developed by the National Institute of Agricultural Research (INIAP) for Andean potato cultivars, ensuring microbial inhibition without inducing phytotoxicity.

### 4.3. Hormonal Treatments and Experimental Design

To evaluate the organogenic effects of cytokinin–gibberellin interactions, a completely randomized design (CRD) with a 3 × 3 factorial arrangement was established [36]. Factor A consisted of three concentrations of 6-Benzylaminopurine (BAP, purity ≥ 98.5%; Plant Cell Technology, Smithfield, UT, USA): 0.5, 1.0, and 2.0 mg/L. Factor B comprised three concentrations of Gibberellic Acid (GA3, purity ≥ 90.0%; PhytoTech Labs Inc., Lenexa, KS, USA): 0.0 (control), 0.1, and 0.25 mg/L. The combination of these factors generated nine distinct hormonal treatments. Each treatment consisted of 20 replicates (glass vessels), with 4 nodal segments vertically inoculated per vessel, totaling 720 explants for the entire experiment. An absolute hormone-free control (0.0 mg/L BAP and 0.0 mg/L GA3) was deliberately excluded from the experimental matrix because the baseline morphogenic response of the ‘Superchola’ cultivar on hormone-free MS medium has been extensively documented in INIAP standard protocols, strictly yielding a single, unbranched stem due to strong apical dominance.

### 4.4. Incubation Conditions and Morphophysiological Assessments

Inoculated vessels were randomized in a growth chamber to prevent microenvironmental biases. Incubation conditions were strictly maintained at 20–22 °C under a 16-h light/8-h dark photoperiod with a photosynthetic photon flux density (PPFD) of 35–45 μmol m^−2^ s^−1^ (approximately 2500–3000 lux) provided by cool-white fluorescent lamps and periodically verified using a digital quantum sensor (model LI-250A, LI-COR Biosciences, Lincoln, NE, USA). Non-destructive kinetic evaluations of shoot multiplication rates were conducted at 7, 14, and 21 days. Destructive morphological and physiological evaluations were executed exactly 28 days post-inoculation. The experiments were conducted over the course of the growing period from day 0 to 28, which strictly aligns with the standard commercial subculture cycle for *Solanum tuberosum* L. Extending the evaluation beyond 28 days introduces confounding variables, such as nutrient depletion and medium senescence, which would artificially skew the biomass and hyperhydricity metrics. Evaluated quantitative parameters strictly focused on dry biomass accumulation to determine true structural growth. To ensure gravimetric accuracy given the minute size of the micropropagated tissues, the four explants within each culture vessel were pooled as a single experimental unit. Biomass parameters were quantified using an analytical balance (model ME54, Mettler-Toledo AG, Greifensee, Switzerland) with a precision of 0.0001 g. For dry weight determination, the pooled samples were subjected to dehydration in a forced-air drying oven (model UF55, MemmertGmbH + Co. KG, Schwabach, Germany) set at 70 °C until a constant weight was achieved. Final biomass values were mathematically expressed as the mean dry weight per explant (n = 20 replicates per treatment). Qualitative physiological disorders, such as basal callogenesis and hyperhydricity (vitrification), were recorded as binary variables (presence/absence), and the Morphological Vigor Index was visually assessed on a standardized 1 to 3 scale based on tissue robustness and structural integrity. To prevent observer bias, this assessment was conducted by an independent researcher blinded to the specific hormonal treatments.

### 4.5. Statistical Analysis

All statistical procedures were executed in the RStudio environment (version 2024.04.2, Posit Software, PBC, Boston, MA, USA) utilizing R software (version 4.3.3, R Foundation for Statistical Computing, Vienna, Austria). Normality and homoscedasticity of the data were initially assessed using the Shapiro–Wilk and Levene tests, respectively. For continuous quantitative variables meeting parametric assumptions (i.e., dry biomass accumulation), data were subjected to an Analysis of Variance (ANOVA), followed by Tukey’s Honest Significant Difference (HSD) test (*p* < 0.05) to determine hierarchical significance among hormonal interactions. Qualitative binary variables (hyperhydricity and basal callogenesis) were analyzed using Generalized Linear Models (GLMs) with a binomial distribution and a logit link function, establishing statistical differences via the Wald test and Tukey’s post-hoc comparisons. Finally, the Morphological Vigor Index, being an ordinal categorical variable, was evaluated through frequency distribution and proportional stacked analysis. Shoot multiplication count data and their two-way interaction effects over time (BAP × GA3) were evaluated using a Generalized Linear Model (GLM) with a Poisson distribution and log link function, followed by Tukey’s post-hoc test.

## 5. Conclusions

The successful in vitro micropropagation of recalcitrant Andean potato cultivars requires a strict balance between maximizing shoot proliferation and preserving structural fidelity and physiological competence. This study concludes that forcing shoot multiplication through cytokinin oversaturation leads to severe morphological degradation, specifically tissue hyperhydricity and basal callogenesis, which ultimately compromise ex vitro survival. However, these physiological disorders can be effectively suppressed through precise hormonal antagonism. The synergistic calibration of high cytokinin levels (2.0 mg/L BAP) with low-dose gibberellins (0.1 mg/L GA3) establishes an optimal morphogenic environment. This specific interaction not only ensures a highly competitive multiplication rate but also maximizes dry biomass accumulation and completely eradicates vitrification. Consequently, implementing this mechanistically balanced protocol in commercial biofactories guarantees the continuous production of morphologically elite, pathogen-free pre-basic seed potatoes.

## Data Availability

The original contributions presented in the study are included in the article/Supplementary Materials; further inquiries can be directed at the corresponding author.

## Supplementary Materials

The following supporting information can be downloaded at https://www.mdpi.com/article/10.3390/plants15193071/s1: Table S1: Analysis of Variance (ANOVA) for Dry Biomass Accumulation at Day 28; Table S2: Generalized Linear Model (GLM) Analysis of Deviance for Shoot Multiplication Kinetics at Day 28.

## Abbreviations

The following abbreviations are used in this manuscript:

ANOVA	Analysis of variance
BAP	6-Benzylaminopurine
CRD	Completely randomized design
DW	Dry Weight
FW	Fresh Weight
GA3	Gibberellic acid
GLM	Generalized linear model
HSD	Honestly significant difference
INIAP	National Institute of Agricultural Research (Ecuador)
MS	Murashige and Skoog (medium)
PPFD	Photosynthetic photon flux density
PPM	Plant Preservative Mixture
