# Supplementary material for: The Synergistic Interaction Between BAP and GA3 Eradicates Hyperhydricity and Enhances Biomass in Solanum tuberosum L. cv. ‘Superchola’ In Vitro Plantlets
Source: Plants (Basel). 2026 Oct 8;15(19):3071. doi: 10.3390/plants15193071 (PMC13666994; doi:10.3390/plants15193071)
Supplement: Supplementary file 1 [file plants-15-03071-s001.zip › plants-4609241-supplementary.pdf]

Supplementary Materials

Title: The Synergistic Interaction Between BAP and GA3 Eradicates Hyperhydricity and Enhances Biomass in *Solanum tuberosum* L. cv. 'Superchola' In Vitro Plantlets.

Authors: Guillermo Alexander Jácome Sarchi, Stalin Aldair De la Cruz Sarchi, Jorge Ivan Mina Ortega, Francisca Hernández, Rafael Todos Santos Martínez

Table S1. Analysis of Variance (ANOVA) for Dry Biomass Accumulation at Day 28

Note: Type III Analysis of Variance for continuous quantitative data (Dry Biomass, mg/explant) evaluated under a 3 × 3 factorial design.

Source of Variation	Sum of Squares	df	F-value	p-value
(Intercept)	0.000126	1	221.9919	< 0.0001
BAP	0.00042	2	37.3665	< 0.0001
GA3	0.00033	2	29.1915	< 0.0001
BAP : GA3	0.000639	4	282.2721	< 0.0001
Residuals	0.000097	171	—	—

Table S2. Generalized Linear Model (GLM) Analysis of Deviance for Shoot Multiplication Kinetics at Day 28

Note: Analysis of Deviance (Type III Wald chi-square tests) for count data (Number of Shoots per Explant) fitted with a Poisson Generalized Linear Model and log link function.

Source of Variation	df	Chi-Square (χ2)	p-value
(Intercept)	1	5.6899	0.0171
BAP	2	1.3027	0.5213

Source of Variation	df	Chi-Square (χ2)	p-value
GA3	2	4.2114	0.1218
BAP : GA3	4	9.7748	0.0444
